# The Buffalo Water Supply

**Published:** 1912-04

**Authors:** 


### The Buffalo Water Supply.

DR. FELL'S article is all the more interesting and welcome because of his vigorous criticism of the editorial in the January issue. We realized at the time of writing, the danger incurred in venturing into deep water by one lacking expert engineering skill and, as stated, our remarks were purely tentative. We were also misled by a detail map which gave an erroneous idea of points of the compass. With these qualifications, however, we are disposed to adhere to the opinions expressed. The controversial points are:

1. The possibility of contamination of the new intake (a) by draft of infection from Buffalo Creek by outgoing vessels; (b) by excrement from vessels in either direction.

2. The practicability of placing the intake above the main commercial channel or vice versa.

3. The feasibility of obtaining reasonably clear water by protective breakwaters.

1. (a) Last year, according to the customs officials, there were more than 3,000 out-bound vessels, not counting tugs, excursion boats, small craft, etc. This was a light year. The commerce on the lake is usually limited to eight months, sometimes less, while the great majority of the passenger business falls in about 100 days from Decoration Day to Labor Day. During the summer months, it is probably an underestimate that two large vessels pass the intake, out-bound, every hour on the average. Often there will be a procession of out-bound vessels. It is a trifle over a mile from the mouth of the creek, about 3,500 feet from the present outer breakwater to the intake. It is not unreasonable to suppose that, by adhesion of infectious matter and possibly by the production of currents, this stream of traffic constitutes an appreciable source of danger.

1. (b) The Crystal Beach boats, last summer carried over a million passengers for round trips. The passenger line of longest distance and least freqency of trips carries about 33,000 passengers a season, one way or the other. For the other lines, excursion and to fairly distant points, no statistics are available. The crews corresponding to the approximately 7,000 entries and exits exclusive of excursion steamers must, at the lowest average estimate, amount to 100,000 individual passages. In the aggregate, about 2,500,000 persons are carried past the intake every season and, on account of the ordinary hours of the main passenger lines, the effects of haste and worry in catching boats and the anticipation of car rides, etc. after leaving them, it is obvious that the discharge of excrement for the period immediately after leaving and before arriving at the dock, is disproportionate to the entire time of passage. It seems reasonable to assume that the excrement of 2,500,000 persons, even for a short space of time, is an element of danger to those drinking the water.

2. While acknowledging that, under existing conditions, it is more convenient and natural for boats to pass above the intake, we can see nothing impracticable, remarkable or peculiar either in an original plan or subsequent alteration aiming to place the intake "up-stream" from a possible source of contamination

The soundings southerly to the intake are from 22 to 25, occasionally 29 or 30 feet, those northerly (down stream) 17, 18, etc. It would obviously be very costly to move the intake but this scarcely seems necessary.

3. So far as our observation goes, roily water moves comparatively slowly into clear water and does not pass beyond dams, break waters, etc., unless these are quite pervious and turned more or less across the current. As already implied, our suggestion of a breakwater at right angles to the present one, was due to a deceptive detail map. Such a breakwater would probably fulfill the purpose but it would have to be unreasonably long. We are inclined to believe that about a kilometer of breakwater in an arc or angle above the intake, allowing free passage of water both to the west and the east, would be more economic and more efficient. Allowing for the dredging of about 5 to 7 feet of silt, the depth of the pool thus formed would be about 10 meters, from mean lake level to bed rock. If the intake is in line with the lower ends of the curved or angular breakwater, the amount of water will be somewhere from one million to sixteen hundred thousand cubic meters (liquid tons) according to the curve or angle employed. Buffalo uses a little over a cubic meter of water per capita per diem, this being a very high figure and one which probably will not be maintained as the population increases. At present, three or four days' water supply could thus be partially impounded. It is obvious that the breakwater should constitute a filter, so as to allow a slow current toward the intake. On the one hand a breakwater so loosely constructed as merely to break the force of the waves, would not prevent the entrance of roily water; on the other hand a practically water-tight wall would merely favor the eddying back of the currents from the extremities of the breakwater. The cost of such a breakwater, at the rate paid for the present one, would be about $310,000.

In conclusion, we want to make one important qualification: that, since the closure of the Bird Island Inlet, Buffalo has usually had very good water and that while we believe every precaution should be taken, Buffalo has already a water supply far superior to most cities!

